# The Berlin Bimanual Test for Tetraplegia (BeBiTT): development, psychometric properties, and sensitivity to change in assistive hand exoskeleton application

**DOI:** 10.1186/s12984-023-01137-4

**Published:** 2023-01-27

**Authors:** Cornelius Angerhöfer, Mareike Vermehren, Annalisa Colucci, Marius Nann, Peter Koßmehl, Andreas Niedeggen, Won-Seok Kim, Won Kee Chang, Nam-Jong Paik, Volker Hömberg, Surjo R. Soekadar

**Affiliations:** 1 Clinical Neurotechnology Laboratory, Department of Psychiatry and Neurosciences, Neurowissenschaftliches Forschungszentrum (NWFZ), Charité-Universitätsmedizin Berlin, Charité Campus Mitte (CCM), Charitéplatz 1, 10117 Berlin, Germany; 2 Kliniken Beelitz GmbH, Paracelsusring 6A, Beelitz-Heilstätten, 14547 Beelitz, Germany; 3 Department of Rehabilitation Medicine, Seoul National University College of Medicine, Seoul National University Bundang Hospital, 82, Gumi-ro 173 Beon-gil, Bundang-gu, Gyeonggi-do 13620 Seongnam-si, Republic of Korea; 4 SRH Gesundheitszentrum Bad Wimpfen GmbH, Bad Wimpfen, Germany

**Keywords:** Tetraplegia, Exoskeleton, Bimanual task performance, Brain–computer interface (BCI), Clinical assessment, Assistive robotics

## Abstract

**Background:**

Assistive hand exoskeletons are promising tools to restore hand function after cervical spinal cord injury (SCI) but assessing their specific impact on bimanual hand and arm function is limited due to lack of reliable and valid clinical tests. Here, we introduce the Berlin Bimanual Test for Tetraplegia (BeBiTT) and demonstrate its psychometric properties and sensitivity to assistive hand exoskeleton-related improvements in bimanual task performance.

**Methods:**

Fourteen study participants with subacute cervical SCI performed the BeBiTT unassisted (baseline). Thereafter, participants repeated the BeBiTT while wearing a brain/neural hand exoskeleton (B/NHE) (intervention). Online control of the B/NHE was established via a hybrid sensorimotor rhythm-based brain-computer interface (BCI) translating electroencephalographic (EEG) and electrooculographic (EOG) signals into open/close commands. For reliability assessment, BeBiTT scores were obtained by four independent observers. Besides internal consistency analysis, construct validity was assessed by correlating baseline BeBiTT scores with the Spinal Cord Independence Measure III (SCIM III) and Quadriplegia Index of Function (QIF). Sensitivity to differences in bimanual task performance was assessed with a bootstrapped paired *t*-test.

**Results:**

The BeBiTT showed excellent interrater reliability (intraclass correlation coefficients > 0.9) and internal consistency (α = 0.91). Validity of the BeBiTT was evidenced by strong correlations between BeBiTT scores and SCIM III as well as QIF. Wearing a B/NHE (intervention) improved the BeBiTT score significantly (*p* < 0.05) with high effect size (d = 1.063), documenting high sensitivity to intervention-related differences in bimanual task performance.

**Conclusion:**

The BeBiTT is a reliable and valid test for evaluating bimanual task performance in persons with tetraplegia, suitable to assess the impact of assistive hand exoskeletons on bimanual function.

**Supplementary Information:**

The online version contains supplementary material available at 10.1186/s12984-023-01137-4.

## Introduction

Due to impaired hand function, individuals with tetraplegia often depend on assistance to perform activities of daily living (ADLs). Patients reported that regaining arm and hand function was their most important goal after cervical spinal cord injury (cSCI) [1, 2]. A common approach to restore hand function in cSCI uses tendon transfer, but this is associated with surgical risks such as infection, tissue damage and anesthetic complications. Moreover, success of tendon transfer highly depends on the quality of muscles and tendons used for such transfer [3, 4]. Another promising approach to restore motor function after cSCI utilizes exoskeletons, i.e., mechanical structures that mobilize the paralyzed limb. In this context, brain–computer interfaces (BCIs) that bypass the lesioned cortico-spinal tract by translating brain activity into control commands have proven particularly promising for intuitive and versatile control of such assistive devices. By using an implantable BCI, severely paralyzed SCI survivors were enabled, e.g., to grasp objects of daily living with a prosthetic arm or exoskeleton [5–7]. Besides implantable approaches that entail the risk of surgical complications, also non-invasive brain-controlled exoskeletons were established, allowing SCI survivors with tetraplegia to independently perform ADLs outside the laboratory (e.g., eating and drinking in a restaurant) [8]. Here, desynchronization of sensorimotor rhythms (SMR) associated with the intention to grasp results in exoskeleton-driven closing motions of the paralyzed hand, while horizonal oculoversions (HOV) opens the hand or stops an unintended closing motion [9]. Since it was shown that repeated use of BCI-driven robotic devices can facilitate motor recovery [10–13], such hybrid brain/neural hand exoskeletons (B/NHE) might play an increasing role for restoration of hand function.

Up to now, the impact of assistive hand exoskeletons on motor function in tetraplegia has only been assessed for unilateral use [8, 14–16]. Many relevant ADLs, however, require the coordinated interplay of both hands, known as bimanual function [17]. Given the importance of bimanual tasks in daily life, it would be important to reliably assess the impact of assistive hand exoskeletons on such bimanual ADLs in tetraplegia. However, there is currently no reliable and valid clinical test available for such purpose. Commonly applied hand function tests, such as the Grasp and Release Test, Capabilities of Upper Extremity Test and Toronto Rehabilitation Institute Hand Function Test (TRI-HFT) do not take bimanual function into account [18–20]. Apart from that, established ADL tests (e.g., the Spinal Cord Independence Measure) are not suitable to assess the impact of assistive exoskeletons since their scoring system solely focuses on level of independence, which, by nature, is biased when applying any assistive device [21].

To overcome this lack of a suitable clinical test, we introduce the Berlin Bimanual Test for Tetraplegia (BeBiTT)—a performance-based test to assess bimanual hand function in tetraplegia that is compatible with the use of assistive exoskeletons. Here, we elucidate the rationale, the design and application of the BeBiTT, and provide evidence for its internal consistency, interrater-reliability, and construct validity across fourteen tetraplegic individuals. To demonstrate the BeBiTT´s sensitivity to assess assistive hand exoskeleton-related improvements in bimanual task performance, the test was applied across ten cSCI survivors who performed the test without (baseline) and with B/NHE assistance (intervention).

## Methods

### Development of the Berlin Bimanual Test for Tetraplegia (BeBiTT)

**Fig. 1:**
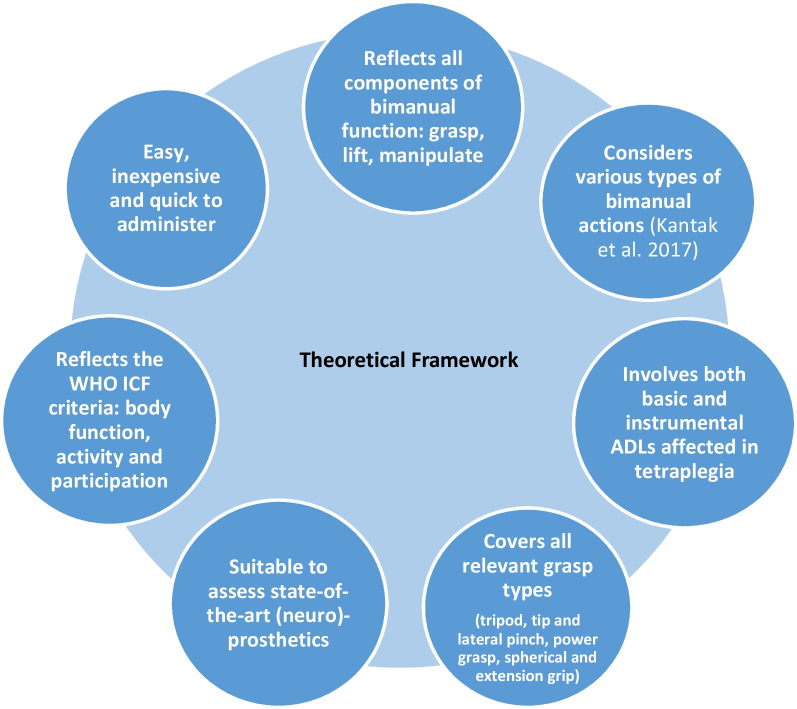
Theoretical framework of the BeBiTT

The BeBiTT is a clinical test designed to assess bimanual task performance in individuals with tetraplegia. It builds on a comprehensive theoretical framework (Fig. 1) and includes conceptual aspects of the Chedoke Arm & Hand Activity Inventory, a well-known test for bimanual performance in the stroke population [22]. The selected test items were selected to cover all grasp patterns relevant in everyday life (tripod pinch, tip pinch, lateral pinch, power grip, spherical grip, and extension grip) and address multiple ADL categories such as eating and drinking, dressing, and personal hygiene. In this way, the participant’s level of functioning can be assessed that reflects the International Classification of Functioning, Disability and Health (ICF) criteria, i.e., body function, activity, and participation. Further, the test items ought to include various types of bimanual actions according to the taxonomy of Kantak et al. [23]. According to the taxonomy, bimanual actions can be divided into symmetric movements involving the use of homologous muscles, and asymmetric movements in which non-homologous muscles are engaged. Further, bimanual actions can be differentiated according to whether each arm/hand intends to achieve an independent goal or whether both hands work together to accomplish a task (common goal tasks). In the latter case, the movements can be executed sequentially (referred to as parallel) or require a cooperative spatio-temporal interaction. While originally designed for stroke populations, we considered Kantak’s taxonomy in the BeBiTT to include a broad range of bimanual actions with varying requirements for coordination.

To find potential test items, a list of 32 bimanual tasks that are impaired in individuals with tetraplegia were generated based on open interviews with affected persons as well as physio- and occupational therapists (both having at least 10 years of working experience with SCI patients). All items were then grouped according to their ADL category (Table 1). With the predefined theoretical framework as a guideline, the most suitable items were selected. Since the BeBiTT was designed to assess the impact of neuroprosthetics and brain-controlled assistive hand exoskeletons, items that involve direct contact to private parts, e.g., in the context of toileting or self-catheterization, was not included. As object manipulation is a core element of bimanual function, items were discarded that did not contain a significant manipulation component such as wheelchair use or transferring and lifting objects only. Moreover, items that appeared too broad to be transferred into practical test tasks like driving a car or playing games were not considered. For practicality in clinical environments, the number of items was kept to a minimum. As a result, 9 items have been chosen for the final version of the BeBiTT, all representing relevant bimanual tasks of daily living that are impaired in individuals with tetraplegia (Table 2). The material needed for the BeBiTT is usually available in clinics and research laboratories and, if not, can be purchased at low cost anywhere in the world (Table 2). Due to its short and easy administration, the BeBiTT normally takes no longer than 20 min to complete. To reliably assess the BeBiTT score, raters are provided with a systematic scoring system (Additional file 1). The scoring system is designed to evaluate the main components of bimanual function in daily activities, i.e., grasping, manipulating, and lifting. For grasping, the scoring system pays special attention whether an active or passive grip is used to accomplish the task. Passive grip refers to the concept of grasping objects with the help of passive tension in the fingers, e.g., by extending the wrist in the so-called tenodesis grasp. While a passive grip is often not very dexterous and powerful, exerting active finger strength contributes to good control and ability to manipulate an object. Accordingly, 2 points are assigned in case an active/firm grip is performed and 1 point is assigned if a passive/loose grip is performed (evaluated for each hand). Moreover, the scoring systems accounts for the level of difficulty the participant experiences during bimanual manipulation and considers the use of compensatory mechanisms. Compensatory mechanisms refer to the use of body parts or functions that are usually not involved in a particular action to compensate for impaired function (e.g., using both hands for a one-handed activity or pinching objects between legs for manipulation). No difficulty in manipulating results in 4 points, slight difficulty in 3 points, great difficulty in 2 points, and 1 point is assigned when solely compensatory strategies are used. Lifting is a good way to evaluate bilateral arm and shoulder function and 2 points are assigned if the bimanual manipulation is performed in a lifted position. Altogether, a maximum of 10 points can be achieved per task (4 for grasping, 4 for manipulating and 2 for lifting). In case a task does not involve any meaningful lifting component, 4 points are assigned for grasping and 6 points for manipulating (Additional file 1). With 9 test items, this results in a score ranging from 0 to 90 points, with 0 points indicating no bimanual function and 90 points indicating unimpaired bimanual function in tetraplegia.

**Table 1:** List of generated items grouped in ADL categories

Eating and drinking	Dressing	Personal hygiene	Preparing food	Communication
Eating with cutlery	Button up trousers	Washing hair	Open food packages	Charge a smart phone
Cutting piece of meat	Close zipper of jacket	Put toothpaste on toothbrush	Open coffee bin	Type on keyboard
Open water bottle (screw top)	Put on trousers	Open toothpaste	Light a cigarette	Sign a contract
Open bottle with (crown cap)	Put on socks	Shaving	Cut a slice of bread	Play games
		Hair styling	Chop vegetables	
		Perform manicure and pedicure		
		Wring out wash cloth		

Transportation	Managing finance	Continence	Housekeeping	
Propel a wheelchair	Take coins out of wallet	Self-catheterization	Rinsing dishes	
Transfer from bed to wheelchair	Take note out of wallet	Toileting	Lift objects from the ground (e.g. laundry basket)	
Drive a car		Light a cigarette	Lift pot or pan

**Table 2:** Berlin bimanual test for tetraplegia

Bimanual task	Material	Grasp pattern	Bimanual action	ADL category
Charge a smart phone	Common smart phone (e.g., iPhone 6, Samsung Galaxy S20) Charging cable	Extension grip (smart phone) Tip pinch (charging cable)	Asymmetric, common goal, cooperative	Communication
Open a water bottle	1-L plastic bottle of water (filled), screwed on by hand	Power grip (bottle) Tip pinch (lid)	Asymmetric, common goal, cooperative	Eating and drinking
Pour glass of water	1-L plastic bottle of water (filled) Water glass (200 ml, empty)	Power grip (bottle) Power grip (glass)	Asymmetric, common goal, parallel	Eating and drinking
Rinse a plate	Dinner plate (approx. 25 cm in diameter) Kitchen sponge	Extension grip (plate) Spherical grip (sponge)	Asymmetric, common goal, cooperative	Housekeeping
Cut meat-like putty	Medium resistance putty resembling the consistency of a piece of meat Knife and fork	Lateral pinch (both hands)	Asymmetric, common goal, cooperative	Eating and drinking
Open toothpaste	Normal 75 ml toothpaste with screwed lid, > 50% full	Lateral pinch (toothpaste) Tip pinch (lid)	Asymmetric, common goal, cooperative	Body hygiene
Apply toothpaste on toothbrush	Normal 75 ml toothpaste with screw lid, > 50% full Toothbrush	Lateral pinch (both hands)	Asymmetric, common goal, parallel	Body hygiene
Take note out of wallet	Common leader wallet 10 Euro or 20 US Dollar note	Lateral pinch (bank note) Extension grip (wallet)	Asymmetric, common goal, parallel	Managing finance
Close zipper of a jacket	Metal zipper in a jacket	Tip pinch (both hands)	Asymmetric, common goal, cooperative	Dressing

### Participants

14 individuals with tetraplegia were recruited (13 male, mean age 48.6 ± 18.5 years) with complete (n = 6; ASIA grade A) and incomplete (n = 8; ASIA grades B–C) cSCI (C4 to C6). Recruitment took place at the University Hospital of Tübingen, the Charité-Universitätsmedizin Berlin, and the Neurological Rehabilitation Clinic Beelitz-Heilstätten (Germany). Participants were selected based on the following inclusion criteria: Age between 18 and 85 years, interval after SCI at least 6 months, lesion height C4–C6, ASIA grade A–C. Exclusion criteria were the following: Consumption of illegal drugs or more than two alcoholic beverages per day, history of severe neurological injuries or conditions other than SCI (e.g., multiple sclerosis, stroke and cerebral palsy), severe medical conditions (e.g., renal/liver/heart failure; malignant tumor disease), serious cognitive impairment and severe spasticity.

All 14 participants took part in the baseline test of the BeBiTT (without intervention). For further validation using an exoskeleton, ten participants (all male, mean age 44.7 ± 14.6 years) with complete (n = 5; ASIA grade A) and incomplete (n = 5; ASIA grades B to D) cSCI (C5 to C6) repeated the BeBiTT using a B/NHE (intervention). All participants were BCI naïve, i.e., they had never taken part in any BCI training or application. Four participants had to be excluded from the intervention due to incapability to wear the hand exoskeleton, arthritic pain, impending hand surgery and skepticism about brain-controlled technology.

### Study procedure

The study protocol complied with the Declaration of Helsinki. Ethical approval was obtained from the Ethical Committee of the Medical Faculty of the University of Tübingen (201/2018BO1). After providing written informed consent, participants were comfortably seated in front of a desk for the BeBiTT baseline assessment. The BeBiTT tasks were explained, and their proper execution was demonstrated by the instructor. The participant was encouraged to perform the tasks as close to the instructor’s demonstration as possible. The participant was reminded of the importance to use both hands and to avoid compensatory strategies if possible. There was no time limit to perform the tasks. The participant could also repeat each task to reach a higher score. After completing the BeBiTT without assistance (baseline), the B/NHE was attached to the participants’ hand and fingers. Then, the BeBiTT was repeated in the exact same manner as in the baseline condition (intervention). The whole session was videotaped, and videos were stored for later review. At the end of the session, all participants were interviewed on the self-care category of Spinal Cord Independence Measure III (SCIM III) and Quadriplegia Index of Function (QIF) and completed a questionnaire assessing safety and feasibility of the BeBiTT.

### Hybrid brain/neural hand exoskeleton (B/NHE)

For the intervention, a wearable hand exoskeleton with nine motor units (HandyRehab from Zunosaki Ltd., Hong Kong) was donned to the participant’s left or right hand, depending on the participant’s choice. Control of the exoskeleton was established by a hybrid BCI, translating EEG and EOG signals into open and close commands. Sensorimotor rhythm event-related desynchronization (SMR-ERD) associated with intended grasping motions was detected by EEG and translated into exoskeleton closing motions. Horizontal oculoversions (HOV) detected by EOG were used to control exoskeleton opening motions or to stop unintended closing [13]. EEG was recorded with a Smarting Mobi system (mBrainTrain, Serbia) and a semi-dry saline-based cap (Greentek Pty. Ltd, Wuhan, China) from 5 conventional recording sites according to the international 10/20 system (C3, Cz, F3, P3, T3 for right hand exoskeleton control, or C4, Cz, F4, P4, T4 for left exoskeleton control). A surface Laplacian filter was applied to reduce signal-to-noise ratio of the target electrodes C3 or C4. Signals were recorded at a sampling frequency of 250 Hz and filtered between 0.1 and 70 Hz with a Butterworth filter. For EOG, two additional electrodes were placed on the right and left outer canthus. The signal amplitude was converted into a bipolar signal and low pass filtered at 1.5 Hz. EEG and EOG signals were processed and classified by a customized version of the BCI2000 software platform (www.bci2000.org) [24].

### Internal consistency, interrater-reliability, construct validity, and sensitivity to change

For assessing internal consistency of the BeBiTT’s test items, Cronbach’s alpha and corrected item-total correlation was calculated using SPSS (v.27) for baseline scores. As suggested by Kline [25], corrected item-total correlation was set below 0.30 for items to be discarded. For assessing interrater reliability, the scorings of the study instructor and three independent raters were obtained. The independent raters were blinded to the participants’ diagnosis and ASIA classification. Raters were given the scoring sheet (Additional file 1) along with a short explanation of the scoring system. They were asked to fill out the scoring sheets for each participant individually and not to discuss the video clips or the assigned scores with each other. Agreement in scores between raters was tested by calculating the intraclass correlation coefficient (ICC) using SPSS (v.27) for both the baseline and intervention. The ICC was calculated based on an absolute-agreement, 2-way mixed-effects model.

To assess construct validity of the BeBiTT, two common ADL tests were assessed by interview: the self-care category of the Spinal Cord Independence Measure III (SCIM III) and the Quadriplegia Index of Function-Short Form (QIF-SF) [21, 26]. While the self-care category of SCIM III and QIF-SF do not assess hand function exclusively, they include many tasks that require bimanual function and are thus suitable to assess construct validity of the BeBiTT baseline scores [27]. The construct validity of the BeBiTT was assessed by computing Pearson’s correlation coefficient between BeBiTT baseline scores and the SCIM III self-care category as well as the QIF-SF.

To assess sensitivity to change between baseline and intervention, a non-parametric bootstrapped paired t-test with 1000 permutations was applied using SPSS. Effect size (Cohen’s D) was calculated. Furthermore, BeBiTT subscores (grasping, lifting, manipulating components) were tested for significance using a bootstrapped paired t-test with 1000 permutations.

## Results

**Fig. 2:**
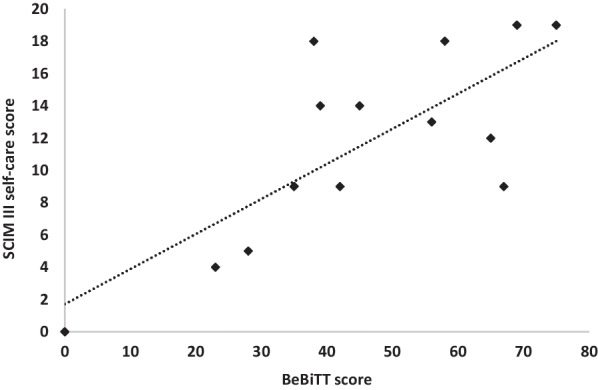
Correlation between of the Berlin Bimanual Test for Tetraplegia (BeBiTT) scores (n = 14) and Spinal Cord Independence Measure III (SCIM III) self-care scores without intervention. BeBiTT scores showed a high correlation with the SCIM III scores (r = 0.77, p < 0.01) evidencing high construct validity

**Fig. 3:**
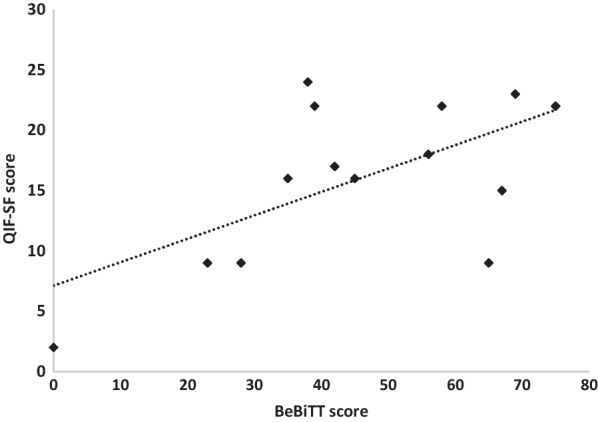
Correlation between the Berlin Bimanual Test for Tetraplegia (BeBiTT) scores (n = 14) and Quadriplegia Index of Function – Short Form (QIF-SF) scores without intervention. BeBiTT scores showed a high correlation with the QIF-SF scores (r = 0.66, p < 0.01) evidencing high construct validity

Participants rated all BeBiTT tasks as feasible and safe. No adverse events or discomforts were reported. In the internal consistency analysis of BeBiTT baseline scores, Cronbach’s alpha reached α = 0.91. All tasks of the BeBiTT positively correlated with the overall score and exceeded the predefined threshold of r > 0.30 (Table 3). For the baseline, raters’ agreement in single values showed an ICC of 0.959 with a 95% confidence interval (CI) from 0.811 to 0.985, F(13,39) = 101.5, and raters’ agreement in mean values showed an ICC of 0.989 with a 95% CI [0.976; 0.996], F(13,39) = 101.5. For the intervention, raters’ agreement in single values showed an ICC of 0.950 with a 95% CI from 0.858 to 0.986, F(9,27) = 116.5 and raters’ agreement in mean values showed an ICC of 0.987 with a 95% CI [0.960; 0.997], F(9,27) = 116.5. Results were significant across all conditions (*p* < 0.001). If removed, the two items ‘pour glass of water’ and ‘apply toothpaste on toothbrush’ would increase alpha by 0.009 and 0.01 respectively (Table 3). To assess construct validity of the BeBiTT, Pearson’s correlation was calculated between the BeBiTT baseline scores (M = 45.7, SD = 20.8) of all participants and scores in SCIM III self-care category (M = 11.6, SD = 6.00) as well as QIF-SF scores (M = 16.0, SD = 6.62). There was a strong correlation between BeBiTT baseline scores and SCIM self-care category scores, r(14) = 0.77, *p* < 0.001 (Fig. 2). Also, BeBiTT baseline scores and QIF-SF scores were positively correlated, r(14) = 0.66, *p* = 0.011 (Fig. 3).

**Table 3:** Internal consistency analysis

Items	Corrected item—total correlation	Cronbach’s alpha if item deleted
Charge a smart phone	0.862	0.891
Open a water bottle	0.840	0.886
Pour glass of water	0.411	0.919
Rinse a plate	0.663	0.900
Cut meat-like putty	0.859	0.886
Open toothpaste	0.937	0.877
Apply toothpaste on toothbrush	0.435	0.920
Take note out of wallet	0.810	0.895
Close zipper of a jacket	0.633	0.903

**Fig. 4:**
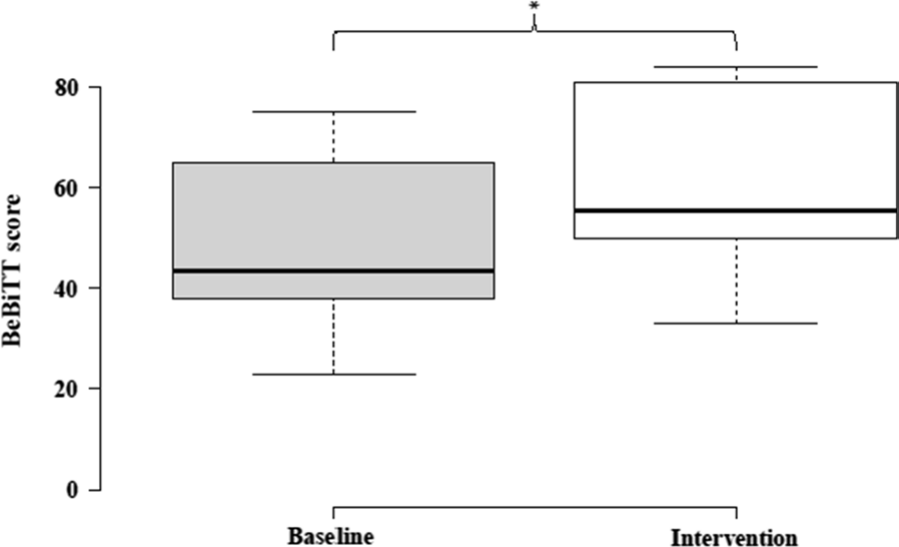
Difference in Berlin Bimanual Test for Tetraplegia (BeBiTT) scores without (baseline) and with use of a B/NHE system (intervention). Centerlines show the medians. Box limits indicate the 25th and 75th percentiles. Upper and lower whiskers illustrate maximum and minimum, respectively. B/NHE application resulted in a marked increase of the BeBiTT score (*p < 0.05) in average across participants (n = 10)

On average, participants improved significantly in BeBiTT score when using the B/NHE system (M = 59.8, SD = 17.4) compared to baseline (M = 48.2, SD = 17.7), *p* = 0.029 (Fig. 4). The bootstrapped (N = 1,000) difference in means was tested for normality and homogeneity of variance and found to be normally distributed (skewness = − 0.38, SE = 0.023; kurtosis = 0.08, SE = 0.047). Bootstrap 95% CI estimate for difference in means did not include zero [6.1;16.4]. The effect size for this analysis (d = 1.063) was found to exceed Cohen’s (1988) convention for a large effect (d = 0.8). Across participants, all components of bimanual function (grasping, manipulating, lifting) improved when using the B/NHE system compared to baseline (Table 4). In lifting, the average score improved by more than 50% from 5.8 (SD = 4.3) to 8.8 points (SD = 4.2), *p* < 0.05. Significant improvement was also shown in grasping from baseline (M = 19.8, SD = 4.8) to intervention (M = 27.3, SD = 5.4), *p* < 0.001. Mean score in manipulating did not improve significantly from 22.6 (SD = 9.9) to 23.6 points (SD = 8.5; *p* = 0.59). On average, all BeBiTT tasks were scored higher with help of the B/NHE system compared to baseline (Table 5). Participants with baseline scores below 40 points showed largest improvement, increasing initial BeBiTT scores by 43.5 to 78.6% when using the B/NHE (Table 6).

**Table 4:** BeBiTT scores for components of bimanual function across conditions

Components	BeBiTT score (baseline)	BeBiTT score (intervention)	Change in score	*P*-Value
Grasping	19.8 ± 4.8	27.3 ± 5.4	7.5 ± 4.0	0.000
Manipulating	22.6 ± 9.9	23.6 ± 8.5	1.0 ± 5.2	0.557
Lifting	5.8 ± 4.3	8.8 ± 4.2	3.0 ± 4.1	0.048

Values are shown as means ± SD

**Table 5:** Improvement in BeBiTT score across all items using an assistive hand exoskeleton

Items	BeBiTT score (baseline)	BeBiTT score (intervention)	Change in score
Charge a smart phone	5.1 ± 1.7	7.2 ± 2.3	2.1 ± 1.4
Open a water bottle	4.4 ± 2.5	5.8 ± 3.3	1.4 ± 1.3
Pour glass of water	5.8 ± 2.8	7.5 ± 2.6	1.7 ± 3.3
Rinse a plate	6.6 ± 2.1	8.9 ± 1.4	2.3 ± 1.8
Cut meat-like putty	5.2 ± 2.3	6.1 ± 2.2	0.9 ± 2.0
Open toothpaste	4.7 ± 2.9	6.4 ± 2.8	1.7 ± 2.3
Put toothpaste on toothbrush	5.5 ± 3.0	5.7 ± 3.5	0.2 ± 3.7
Take note out of wallet	7.7 ± 1.6	8.2 ± 1.9	0.5 ± 2.0
Close zipper of a jacket	3.5 ± 2.9	4.0 ± 2.6	0.5 ± 2.6

Values are shown as means ± SD

**Table 6:** BeBiTT scores for components of bimanual function across conditions and participants

Participant	ASIA Impairment Scale	Level of injury	BeBiTT score (baseline)	BeBiTT score (intervention)	Improvement (%)
ID_1SCI	A	C5/6	69	84	21.7
ID_2SCI	C	C5	45	60	33.3
ID_3SCI	A	C6/7	42	45	7.1
ID_4SCI	B	C6/7	38	57	50.0
ID_5SCI	A	C5/6	28	50	78.6
ID_6SCI	D	C5	65	51	− 21.5
ID_7SCI	D	C4/5	67	–	–
ID_8SCI	A	C5/6	56	–	–
ID_9SCI	B	C4/5	39	54	38.5
ID_10SCI	B	C5/6	75	83	10.7
ID_11SCI	A	C4/5	0	–	–
ID_12SCI	A	C6/7	58	81	39.7
ID_13SCI	A	C5	35	–	–
ID_14SCI	B	C5	23	33	43.5

## Discussion

We have introduced the BeBiTT as a broadly accessible test to evaluate bimanual task performance after cSCI and have validated it across 14 tetraplegic individuals. Our results show that the BeBiTT is a feasible, reliable, and valid tool to assess bimanual task performance in tetraplegia. Cronbach’s alpha (0.91) exceeded the threshold for clinical instruments (> 0.9) defined by Nunnally [28], and documented excellent internal consistency. Only two items (Pour glass of water and Apply toothpaste on toothbrush) would slightly increase alpha if deleted. Although this would marginally increase the internal consistency of the BeBiTT, we decided not to discard the two items given the already excellent alpha score of 0.91 with all items included and the already small number of items. Scores of each item positively correlated with the total score (r > 0.30), further underlining the internal consistency of the test. Interrater reliability, calculated according to Koo and Li [29], was excellent (ICC > 0.9) for both baseline and intervention, indicating that the BeBiTT can reliably assess bimanual task performance with or without the use of assistive tools such brain-controlled exoskeletons. Support of the BeBiTT’s construct validity was provided by a strong positive correlation between baseline scores of the BeBiTT and the SCIM III self-care category as well as the QIF-SF.

**Fig. 5:**
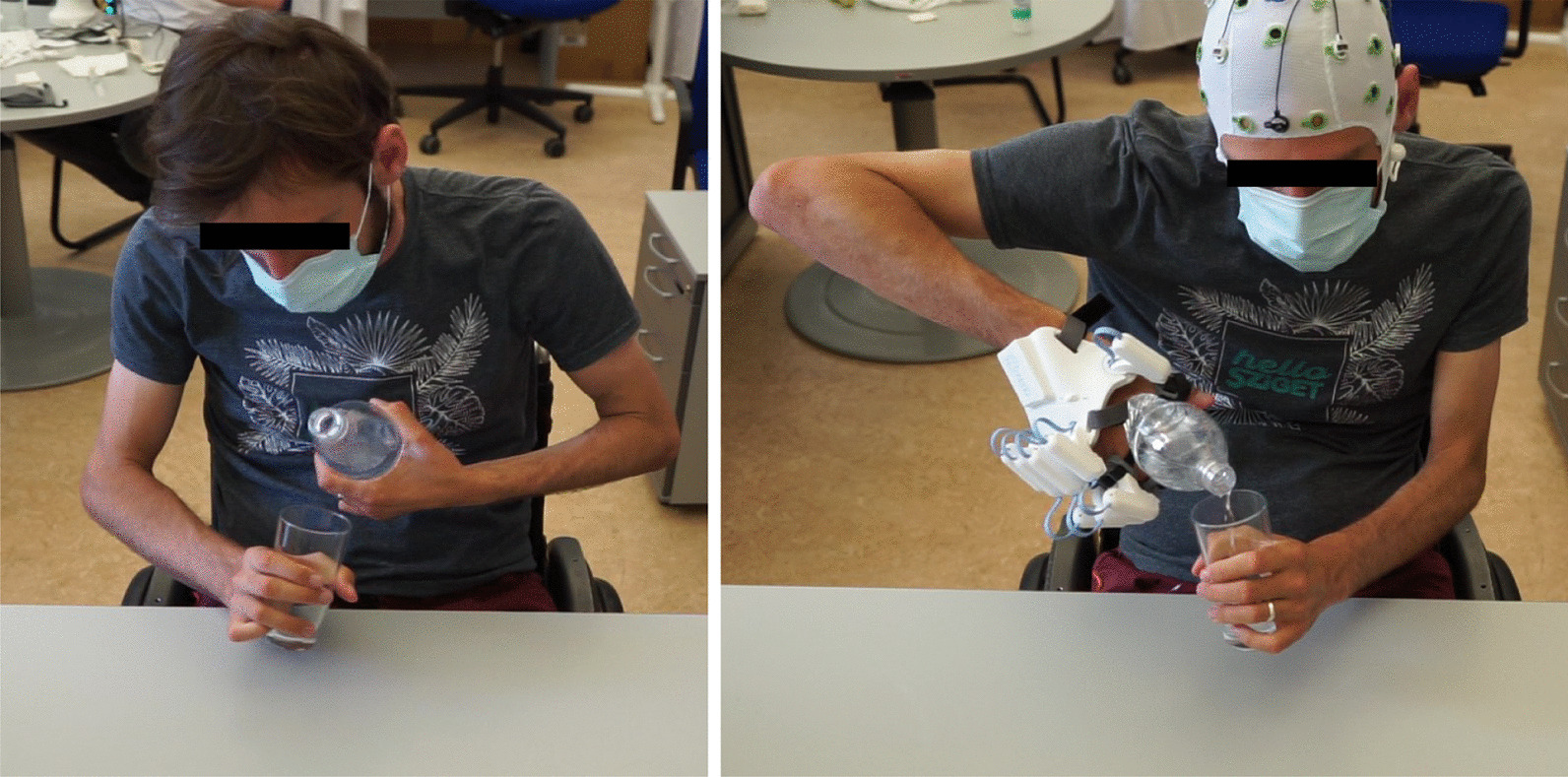
Participant during baseline (left) and while using a brain/neural hand exoskeleton (B/NHE, right) for the BeBiTT item ‘Pour glass of water’. Without the B/NHE, the participant was unable to securely hold the bottle, so that he had to press the bottle against his torso to stabilize it (left). The B/NHE enabled the participant to firmly grasp and lift the water bottle, allowing him to complete the task without difficulties (right). Control of the B/NHE was established by a hybrid BCI, translating electroencephalographic (EEG) and electrooculographic (EOG) signals into hand opening and closing movements

Besides validating the BeBiTT’s feasibility, reliability, and validity, we demonstrate its sensitivity to assess improvement in bimanual task performance related to assistive tools, such as a B/NHE. While individuals with tetraplegia usually perform the (passive) tenodesis grasp that does not provide a strong grip necessary for ADLs, assistive hand exoskeleton control allowed for active movements and sufficient grip strength in bimanual tasks, resulting in increased scores in grasping. Moreover, the hand exoskeleton improved lifting scores across all participants as it provided a secure and firm grip that did not lose stability and strength, enabling participants to securely perform bimanual tasks in a lifted position (Fig. 5). Only in manipulation, no significant improvement was achieved. This could be due to the limited capacity of the applied exoskeleton to perform various grasp types, or limited routine of the users with handling such assistive device. While some participants had difficulty performing fine manipulations with the exoskeleton due to its unwieldiness, others were able to handle the exoskeleton better which resulted in improved manipulation. This observation is supported by the large interindividual variability in manipulation scores as expressed in a high standard deviation. Overall, the performance of the BeBiTT improved with B/NHE use across all participants.

Participants with a baseline BeBiTT score below 40 showed to benefit the most from such intervention. High effect size further exemplified the BeBiTT’s ability to detect enhancement in bimanual function through assistive hand exoskeleton use. By presenting the BeBiTT’s sensitivity to change, we demonstrate the impact of B/NHEs on bimanual task performance in a systematic and replicable way.

While the BeBiTT was originally designed to assess the impact of neurotechnology-based assistive devices, it can also be applied to evaluate alternative approaches that aim at restoring hand function after SCI, e.g., assessing the effect of tendon transfer or functional electric stimulation (FES) on bimanual function in individuals with tetraplegia. The BeBiTT does not only allow to monitor individual therapeutic success but also facilitates the comparison between different training approaches in occupational therapy and physiotherapy. In addition to its use in individuals with tetraplegia, the BeBiTT can be used as a template for assessing bimanual function in other patient groups, such as stroke survivors [12, 13, 30, 31], although adjustments may need to be made according to the population’s level of impairment. The BeBiTT might be also a useful tool to evaluate recovery of bimanual task performance secondary to neuroplasticity, e.g., triggered by repeated use of assistive exoskeletons [10, 32]. Generally, we believe that bimanual function should be given more attention in upper limb rehabilitation, considering the relevance of bimanual tasks in daily life. By providing a reliable and accessible tool for the assessment of bimanual function, we hope to facilitate future research into this direction.

While some ADL categories such as eating and drinking are well represented in the BeBiTT, other categories are only represented by one item to reduce the time of administration and practicality. Representation of these less represented ADL categories could be improved by including more items but would also substantially increase the time of test administration. While psychometric evaluation of the BeBiTT showed excellent results, the reported findings must be further validated across larger clinical studies to foster its broad clinical acceptance. In addition, it is unclear whether the reported improvements in bimanual task performance using brain-controlled exoskeletons generalize to larger clinical populations. Although the present study involved the highest number of study participants in which a BCI-driven hand exoskeleton was used to assess bimanual task performance in tetraplegia to our knowledge (n = 10), more data is necessary to account for demographic factors such as time since injury, ASIA classification and lesion height. In the present study, there was also no control for confounding variables such as sequence effects or fatigue. Previous studies showed that fatigue and lack of concentration can affect BCI performance [33]. Since the baseline was assessed first, this could have caused learning effects, positively influencing the outcome of the intervention. In contrast, fatigue might have affected the outcome into the opposite direction. Moreover, the participant’s capability for BCI control was not directly assessed (e.g., by determining false-positive or false-negative control commands), but indirectly through functional improvement in the BeBiTT. Poor exoskeleton control could explain why one participant performed worse in the intervention compared to baseline. Here, it is conceivable that more routine with handling assistive hand exoskeletons would improve manipulation abilities as measured by the BeBITT. In the present work, B/NHE control was only applied to one hand. Future studies should investigate how bimanual task performance is affected when users wear bilateral B/NHE as introduced by Nann et al. [34], providing more information of the BeBiTT's responsiveness to neurotechnology-based interventions.

## Conclusion

This study shows that the BeBiTT is a reliable and valid test to assess bimanual task performance in tetraplegia, e.g., to evaluate the impact of a brain-controlled hand exoskeleton. Availability of the BeBiTT closes an important gap in the clinical evaluation of upper limb motor function after SCI that, so far, did not sufficiently addressed bimanual task performance and compatibility of the test with the use of assistive tools. Assessing the BeBiTT takes less than 20 min and can easily be incorporated in any clinical and research environment.

## Supplementary Information

**Additional file 1.** BebiTT Scoring Sheet.

## Data Availability

The videotapes of participants analyzed in the current study are available from the corresponding author on reasonable request. All other data generated or analyzed during this study are included in this published article.
