# Supplementary figures and images for: The Berlin Bimanual Test for Tetraplegia (BeBiTT): development, psychometric properties, and sensitivity to change in assistive hand exoskeleton application
Source: J Neuroeng Rehabil. 2023 Jan 27;20:17. doi: 10.1186/s12984-023-01137-4 (PMC9881328; doi:10.1186/s12984-023-01137-4)

**
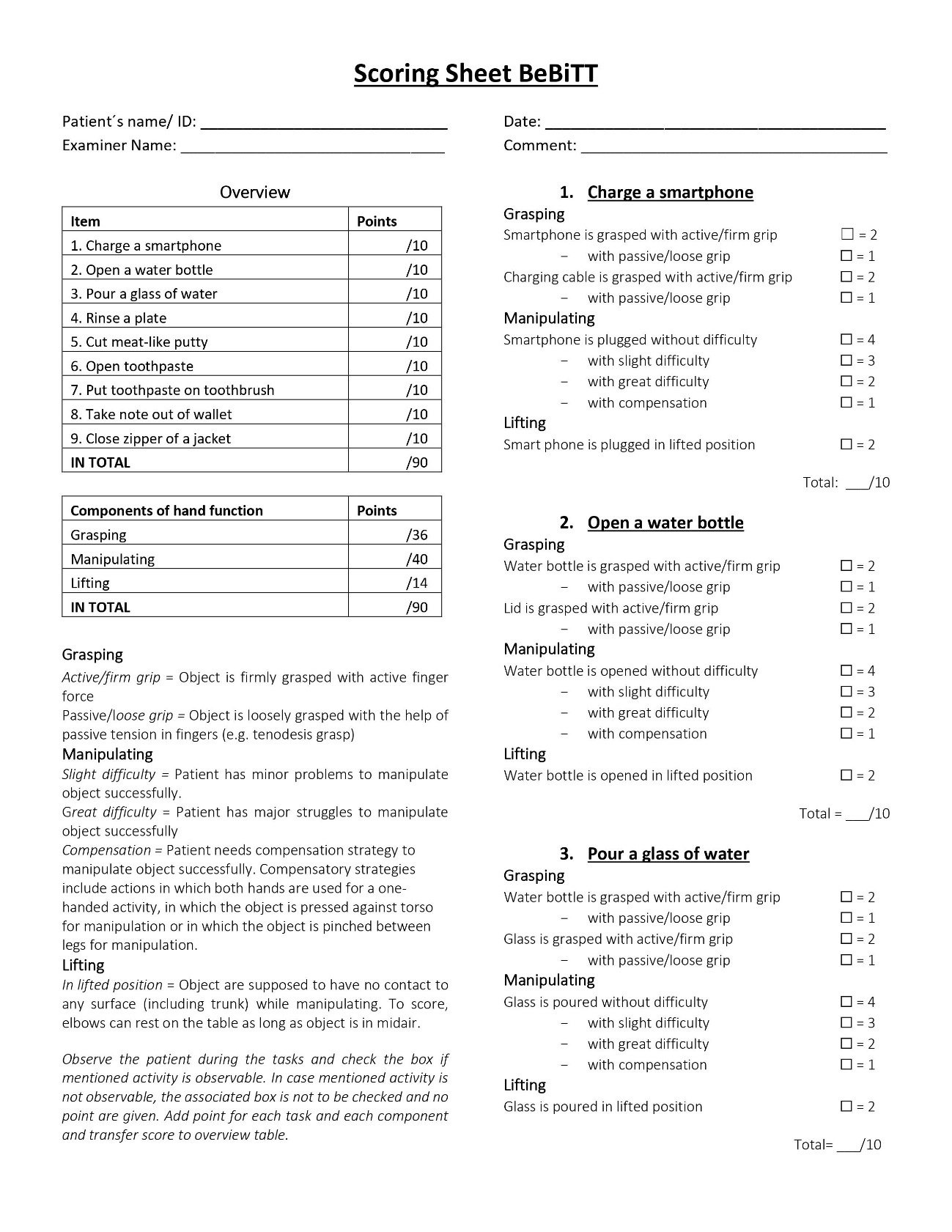
Supplementary material**


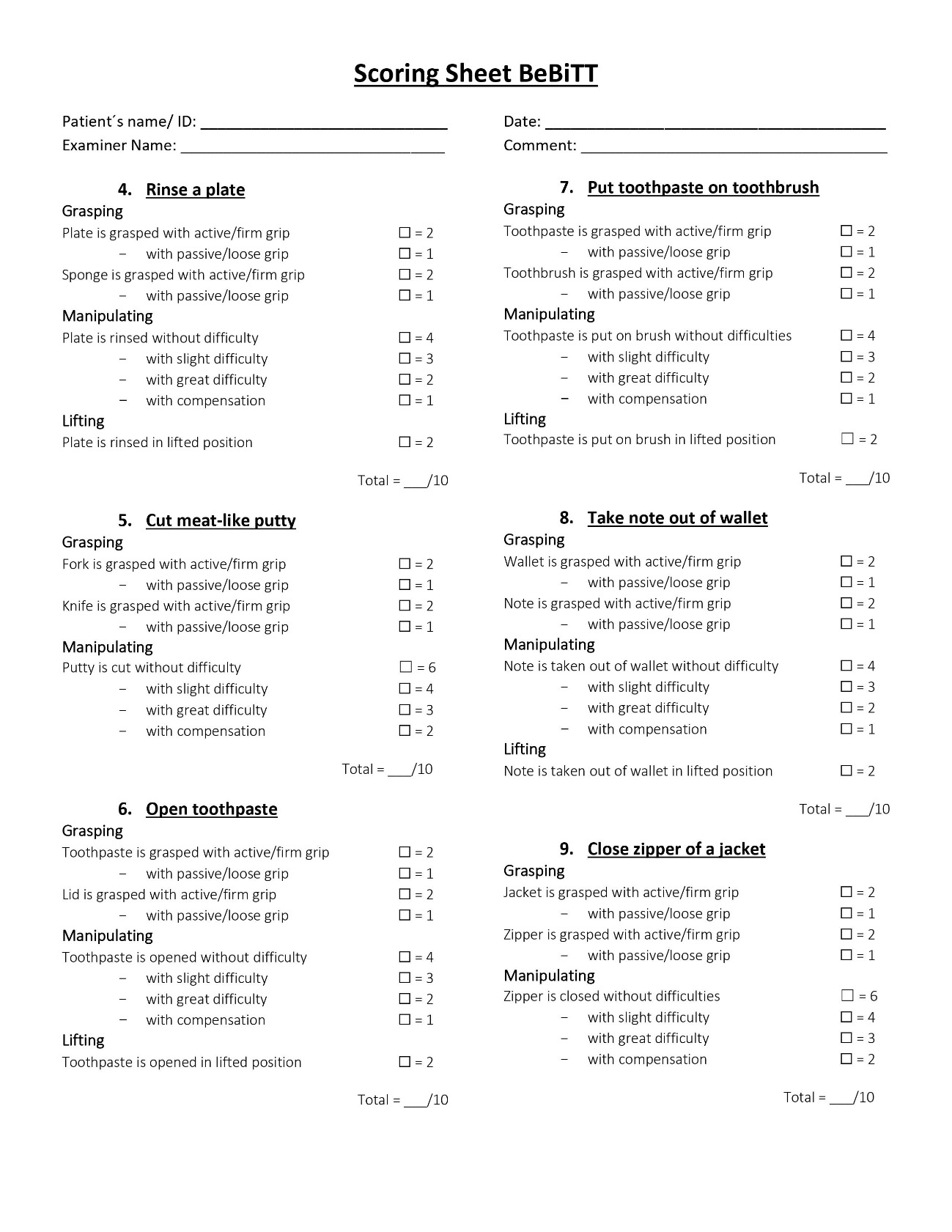

Supplement: Supplementary file 1 — Additional file 1. BebiTT Scoring Sheet. [file 12984_2023_1137_MOESM1_ESM.docx]
